# A Qualitative Case Study on Influencing Factors of Parents’ Child Abuse of North Korean Refugees in South Korea

**DOI:** 10.3390/healthcare9010049

**Published:** 2021-01-05

**Authors:** Wonjung Ryu, Hyerin Yang

**Affiliations:** The Center for Social Welfare Research, Yonsei University, Seoul 03722, Korea; wjryu514@gmail.com

**Keywords:** North Korean refugees, child abuse, acculturative stress, parenting self-efficacy, qualitative case study

## Abstract

The purpose of this study is to investigate the influencing factors of parental child abuse by North Korean refugees who are living in South Korea. In-depth interviews were conducted with five parents who escaped from North Korea. The study identified three categories of factors impacting child abuse: the weakening of family functions from past experiences before and after defection, the stress of adapting to the culture of an unfamiliar society, and low parenting self-efficacy. North Korean parents suffered from emotional and functional crises from past traumatic events and, at the same time, experienced additional acculturative stress as a “minority” after entering South Korea, even as they continued to deal with Maternal Parenting Stress. These complex factors have been shown to lead to child abuse in migrant societies. This study contemplated the context of child abuse through specific examples. The results could provide thoughtful insights into child abuse among migrants and refugee parents, and provide evidence-based intervention plans for its prevention.

## 1. Introduction

North Korea’s communist regime has been committing human rights violations throughout its three-generation-long reign of terror. Even at this moment, many North Koreans are in various kinds of anti-human-rights situations, enduring violence, torture, hunger, and social control. For this reason, many try to escape every year. South Korean society refers to those who have escaped from North Korea as ‘North Korean refugees’ (‘NK refugees’).

The number of NK refugees who have defected from the North and settled in the South is now at about 37,000 [1]. The entry of NK refugees to South Korea has gradually increased since the 1990′s when the severe economic crisis began in North Korea, with more than 1000 NK refugees still entering South Korea every year [1] (p. 1). As the number of NK refugees has increased over the past two decades, a new type of family, termed a “North Korean family” (an ‘NK family’) has been created in South Korea. While the correct figures have not been aggregated recently, the pattern of escaping from North Korea with ‘family units’ has been increasing. And the fact that 77.3% of NK refugees in South Korea in their 40′s or younger [1] (p. 1), thus in their childbearing years, implies that NK families could gradually become a meaningful family type in South Korean society. It is easy to erroneously believe that all members of North Korean refugee families are actually from North Korea, but by looking deeper into the families, it becomes clear that their countries of origin vary, including people from North Korea, China, and South Korea, due to human trafficking, separation, reunion, and remarriage in the process of defection. This means that many NK families have low family bonds and cohesion, which results in frequent family conflicts or domestic violence.

In fact, according to a recent survey, 49% of NK refugees have physically abused their children, while 57.7% of them have ever committed emotional abuse [2]. This figure is about three times higher than the ratio of South Korean parents’ abuse identified in the same survey. This clearly shows NK refugee vulnerability around the issue of child abuse.

The problem of child abuse is serious not only among NK refugees’ families but also in other refugee families in Western society. Previous research has shown that child abuse in refugee families is more prevalent than among ordinary immigrant families, and they pointed out that it can occur in different patterns [3,4].

A multitude of services and interventions are being developed as social concerns over refugee family abuse gradually increase, but NK refugees have another different character compared to other refugees. Therefore, understanding these families’ specific nature and environments is very important to solve the problem of child abuse in NK refugees’ families. Unlike other refugees, NK refugees experienced fear from the reign of terror in North Korea, and they have been forced into particular actions and beliefs in a ‘completely controlled society’ for the past 70 years. Examples include forcing a belief that denies capitalism or providing socialist education based on loyalty to the leader. After entering South Korea, they face additional pressures, having to adapt quickly to a new society, experiencing a lot of stress due to job insecurity, economic difficulties, a lack of support, discrimination, and exclusion from South Korean society. They are also unstable in their roles as parents or have low confidence in their parenting abilities [5]. Such transitional experiences and processes make the risk of child abuse more higher.

The South Korean government and many NK refugee support organizations are actively looking for social services and interventions to prevent child abuse overall, but they currently lack a concrete approach to ‘NK families.’ The interventions for preventing child abuse should be implemented based on an understanding of the specific situations NK refugees’ families face because this is a good way to increase the effectiveness of prevention strategies and mitigate the recurrence of abuse. Therefore, this study, based on the qualitative interviews with NK refugee parents, identifies specific reasons why child abuse occurs within their family context, and ultimately seeks to examine measures that could prevent child abuse.

### 1.1. Characteristics of NK Refugee Families Living in South Korea

In order to understand the child abuse behaviors of NK refugees, one must first consider the characteristics of NK refugees’ families and the main characteristics of their distinctive environment.

First, NK refugees experience serious trauma during their defection. There are three main routes of defection to enter South Korea: ① via China; ② via East Asian countries through China; or ③ via Mongolia through China. Throughout the ordeal, a large number of NK refugees confront life-and-death situations. According to a previous study, 96.5% of NK refugees endured more than one traumatic event [6]. The study found that they experienced incidents repeatedly, which rarely happens to ordinary people in their lifetime [7], with trials encompassing food shortages, labor camp imprisonment, torture and beatings, human trafficking, and having to avoid arrest by the secret police. Unfortunately, they also experienced many traumatic events after arriving in South Korea because the social, economic, and cultural environments between the two countries have diverged since their division more than half a century ago. As the traditional culture in which these North Koreans had lived with is, for the most part, not accepted by South Korean society, they repeatedly face social discrimination and stigma in their daily lives caused by acculturative stress [8,9].

Second, their family functions are weak due to differences in family culture between the South and the North, especially in family-related experiences. The patriarchal family culture is dominant in North Korea, where male-dominant aspects of family authority and role distribution loom large [10]. They also have a strict parent-child relationship [11]. This is different from South Korea’s family culture, which emphasizes a democratic family lifestyle and gender equality [12].

These differences cause confusion among individuals and family members, weakening the family function and creating difficulties in the child-rearing process. In addition, many North Korean defectors experience a break with their families still in the North. Because NK refugees usually bring their remaining family members to the South after some of them escape first, due to strict surveillance by the NK secret police. In this process, many children who experienced separation from their parents suffer from physical and mental dysfunction, and have emotional problems such as anger, sadness, and so on [7] (p. 2). In addition, if a family member is known to have entered the South, the remaining family members in the North will be subjected to serious crises, including interrogation, torture, and detention in labor camps from the NK authorities [7] (p. 2). In this way, their special experiences in defecting from North Korea cause complex emotions and conflicts within their family, which consequently weakens the family’s functionality, negatively affecting the process of adapting to South Korea.

### 1.2. Influencing Factors of Child Abuse in Migration Family

#### 1.2.1. Acculturative Stress and Child Abuse

NK refugee parents encounter difficulties not only in raising their children but also in settling in an unfamiliar society. These have an important impact on both the growth and development of their children and on the parents themselves [13]. According to the acculturation model, ‘acculturation’ is a cultural and psychological change process that migrants experience when they engage with a new society [14], and many immigrants face a number of stressful situations in adapting to a new country [15,16].

In general, the more stress parents experience, the more likely they are to lose confidence in their parenting [17]. The parent’s stress is closely related to child abuse, in that the stress causes them to choose punitive methods over more positive ones as their main parenting style [18,19]. Migration and refugee studies also show that many parents experience acculturative stress regardless of whether they prepared for the migration situations in advance or not [20,21], which means there is a strong correlation with child abuse by parents in immigrant families [21] (p. 3), [22].

NK refugees’, vulnerability to stress is extreme because they face dramatic risks, including threats of forced repatriation during their escape. As previously mentioned, they are placed in very stressful environments even after resettlement, since North and South Korea have wide differences in their societies--social, cultural, political, educational, religious, etc. According to Park [5] (p. 2) study on the parenting of North Korean defectors, the stress of the NK refugee parents was found to be the main reason for dysfunctional parenting. NK refugee parents are not only exposed to constant stress, but they feel a lot of pressure related to their children’s care and tend to negatively evaluate, criticize, and neglect their children [13] (p. 3). As such, the stress of NK refugee parents can be closely linked to child abuse, and the problem of child abuse can become severe, as they lack opportunities to relieve stress and also lack access to protective resources within South Korean society.

#### 1.2.2. Parenting Self-Efficacy and Child Abuse

Parenting self-efficacy refers to the confidence, belief, and ability in the parenting role that parents obtain as they raise their child [23]. This is important for parents to be able to successfully cope with the problems of child-rearing. In general, parents with low parenting efficacy tend to choose a controlling and punitive parenting style [24,25]. Conversely, parents with high parenting efficacy tend to choose a receptive attitude and a non-punitive parenting style [26,27]. These characteristic styles show close relevance to child abuse.

A study by Lee, et al. [13] (p. 3) showed that NK refugee parents had significantly lower parenting efficacy than South Korean parents, and that their confidence in parenting was extremely diminished. In fact, many NK refugee parents also feel they lack the ability to educate their children because the education methods and content between the two Koreas have changed during the period of division [7] (p. 2). Especially in the unusual context of ‘migration’ and ‘transition’, they are less confident about maternal roles and parenting abilities, defined as child-rearing effectiveness [5] (p. 2), [9] (p. 3), [13] (p. 3). Specific research such as this is essential in that unstable parenting efficiency of NK refugees’ parents leads to an inconsistent and abusive parenting style, which can lead to various forms of abuse.

## 2. Methods

### 2.1. Participants

#### Characteristics of study participants

Charateristics of study paricipants are as follows (Table 1). This study was conducted to identify the factors of abuse through an in-depth study of NK refugee parents. Therefore, the subjects of this study were described as ‘NK refugee parents who are raising elementary-, middle-, and high-school children’, with a total of five NK refugee parents participating. The period of residence in South Korea ranged from two to six years. The ages of the participants included one people in their 20s, one people in their 30s, two people in their 40s and one person in her 50s. All subjects, except for participant E, lived together with their spouses. The number of children is a minimum of one and a maximum of two.

### 2.2. Data Collection and Analysis

Due to the characteristics of subjects, convenience sampling in schools for NK refugee children was conducted.

Participants were recruited from three schools in Seoul and Incheon, and the study was conducted with parents who volunteered to take part in the study. In-depth interviews were conducted between July and November 2018. As most of them were engaged in irregular economic activities, one-to-one or one-to-many interviews had to be done based on the participants’ circumstances; each participant was interviewed three times. The qualitative interview was conducted in a comfortable atmosphere. The interview was conducted by one interviewer, but the data analysis process was cross-validated with co-author. Before the in-depth interview, the interviewer had enough time to form a rapport with the participants, and the in-depth interview questions were conducted with semi-structured questionnaires such as ‘How is your relationship with your child’ and ‘What does your child mean to you?’ The interviewer added meaningful questions to suit the interview situation. The transcript was prepared within 2–3 days of the interview, and nonverbal elements such as facial expressions and tone found during the interview were additionally recorded. IRB approval was obtained from the ethics committee of the Yonsei University Institute Review Board (IRB No. 7001988-201810-HR-408-04.

Qualitative case studies were selected as the research method. Qualitative case studies are a way of identifying relationships within specific contexts based on an understanding of cases (Stake, 1995). This method was considered appropriate for this study because it seeks to build an in-depth understanding of the experiences of NK refugees living in South Korea.

The data analysis employed the analytic strategies of ‘Categorical Aggregation or Direct Interpretations’ presented by [28] Stake, with meaningful statements from the recorded data sorted into common themes and aggregated, thereby creating similar categories based on links between them. Triangulation was conducted among the researchers to enhance the validity of the analysis, with expert advice consulted in an effort to increase the accuracy of the content.

## 3. Results

The results analyzed through the qualitative research are as follows (Table 2).

### 3.1. Diminished Individual & Family Functions Due to the Past

#### 3.1.1. Unstable Attachment between Parents and Children Due to Long and Repetitive Separation

The main way in which the subjects entered into the South was the ‘pre-entry of parents and then the entry of children’. Most of them experienced separation from their children during the process of defection. The period of separation varied from one to ten years. For them, it was the best choice—to bring their children South after establishing a safe escape route and settling down. Unfortunately, such a long-term separation weakened the attachment between the parents and their children. During the time they were separated, the children tended to feel betrayed, had thoughts that they wouldn’t be able to join their parents, and experienced both longing and resentment toward their parents, which negatively affected parent-child relations after the reunion.

*“At that time, I chose that way—the pre-entry of parents and then of their child—for my kid, but I guess it was a hard time. Whenever there’s a conflict between us, the kid tells me about the experience at that time, getting mad at me.”*

[Participant A]

*“I finally got a chance to meet my kid after four years, but he didn’t want to talk to me for three months. I tried to have a conversation but he almost refused… [skip] He kept behaving in that way, which made me stop trying to put effort into him.”*

[Participant E]

#### 3.1.2. Emotional Disorders of Parents Caused by Traumatic Events

Most of the participants in the study experienced trauma in the process of escaping. One of them witnessed their daughter being shot to death during the escape; another was tortured at an NK detention facility after being arrested. Other participants also experienced a serious life crisis during that time. They experienced severe after-effects such as depression, isolation, and PTSD from past experiences, and it directly affected their parenting. Participant B confessed that she struggled with the trauma and couldn’t do anything for three months, which eventually made her abuse her child badly. Participant D said that after the dangerous defection, she became aggressive and her sense of victimization became very severe. This emotional condition had a negative impact on child-rearing, with her becoming sensitive to the child’s words and behaviors, and becoming a person who easily got mad at her child’s tiny mistakes.

*“The previous memories repeatedly coming to my mind, I couldn’t do well in my daily life for several months. I felt depressed, sad, lonely…and I didn’t even want to see my child playing. So I sometimes hurt him.”*

[Participant B]

*“Since past trauma experiences, I’ve become angry and impulsive. So I was more likely to be mad at my kid, hurting him by talking in a bad way.”*

[Participant D]

### 3.2. Acculturative Stress in South Korean Society

#### 3.2.1. Stress Expression and Transfer during the Child-Rearing Process

NK refugee parents were found to be very stressed by South Korean society. They were filled with depression and fury by the discrimination, exclusion, and tendency to recognize them as ‘strangers’ that they faced in South Korean society. This situation made it difficult for them to think normally and exacerbated even small problems in the child-rearing process. Participant E, whose defection motivation was for her ‘children’s future,’ transferred the stress and the related emotions that occurred during the adaptation process onto her children. Sometimes she threatened to send them back to China. In this way, the children became the object of their parents’ anger. Participant C said that excessive stress brought about in the process of adapting caused a sense of helplessness in her life, which led to the neglect of her children.

*“It’s not easy to live in South Korea being treated as a ‘stranger’ and I ended up getting angry at my child. Thinking of it, it was not a big deal… (skip) but I said a lot of acrimonious things to him. When I was angry, I even told him that I’ll send him back to China, and one day he said it really hurt him.”*

[Participant E]

*“The pressures… I gave all to my child. Even if my child was hungry, I left him alone.”*

[Participant C]

In the case of participant A, the more stressed she felt in South Korean society, the more obsessed she was with her child’s performance and success, recognizing ‘children’ as the only factor to get out of the stressful reality. This led to excessive interference and control of her children’s behavior, and to her raising them under strict discipline and with corporal punishment.

*“Well, I thought living here, in South Korea, after all the tough times, would be worth it only when my kid succeeds in his career. So, I think I’ve become more obsessed with him.”*

[Participant A]

#### 3.2.2. Conflicts Due to Differences in Adaptation Levels between Parents and Children

NK refugee children have turned out to be relatively less stressed and faster at adapting to life in South Korea than their parents. These differences in adaptation level are found to cause differences in values between parents and children, and to eventually deepen the conflicts between parents and children. In the case of participant C, her children, who adapted quickly to South Korea, had a high level of comprehension in democracy, human rights, and equal family culture. And when facing conflicts, parents felt ignored or challenged to exert parental authority over their children who claim these values. These feelings have led parents to more strict discipline and corporal punishment than ever. Some North Korean parents also thought that abuse had the effect of weakening their children’s rebellious behavior, which led them to stick to abusive parenting or to gradually increase the level of their abuse.

*“They’ve already changed quickly after entering South Korea. One day, my child talked back to me about equality and human rights, and I don’t now why… but I felt unpleasant, so I scolded him.”*

[Participant C]

*“As my child has grown up, I thought that my authority had become ignored and challenged by him. Sometimes I hurt him physically as I thought he’d keep ignoring me if I don’t scold him.”*

[Participant B]

### 3.3. Decrease in Parenting Efficacy

#### 3.3.1. Confusion in the Role of Parents before and after Migration

NK refugees experienced confusion in their parenting roles in the process of migrating to a new society, and they felt frustration around bringing up their kids. According to participant D, whenever their parenting behaviors learned in North Korea were considered a bad parenting style in South Korean society, they felt confused about their role as parents. Excessive interference and criticism form South Korean society reduced their confidence in parenting further. This low parenting efficacy has caused child abuse.

*“In some cases, the parenting beliefs that I have had from North Korea was considered a child abuse act in South Korean society. It was confusing, so I tried to change the way of raising my kids many times, but it didn’t help but just confused my kids too.”*

[Participant D]

#### 3.3.2. Lack of Parenting Information

The shortage of parenting information was a common characteristic among NK refugee parents. Since there are no parenting education programs in North Korea, NK refugee parents enter into the South without knowledge relating to parenting. South Korea currently has a variety of parental education programs, but lacks customized parenting education programs for NK refugees. Aside from this, it is very difficult for those who have to engage in irregular economic activities to participate in education programs. The NK refugee parents also lack social support resources, so they do not have supporters to turn to and ask for help when raising children is difficult. Thus, they gradually lose confidence in their role as parents. The problem is that in such cases, they tend to choose a corporal punishment-oriented parenting style, which is familiar to them from their North Korean culture.

*“I heard that there are many parenting education programs in South Korea, but it doesn’t really help us as it is more important for us to make money than learning from the program. So… I raise my child by how I learned in North Korea.”*

[Participant A]

In sum, NK refugee parents experience emotional crises and stress in the process of defection, which weakens the functionality of the family or individual. These risk factors affect their parenting style. The three meta-categories identified in this study as single factors directly affect the way children are raised and are associated with abusive behavior. This study confirmed that the factors are interrelated and induce child abuse. Moreover, the absence of protective factors, such as support resources and parenting information in South Korean society were revealed to make their abusive behavior tendencies more serious.

## 4. Discussion

This study has important academic value in that it has identified the factors of NK refugees’ child abuse behaviors within the specific context of migration. The results of this study show that, from their experiences during the process of defection, NK refugees have weakened their personal and family functions, which negatively affects their attachment and family bonding, thereby indirectly increasing the risk of abuse. Specifically, North Korean parents who experienced traumatic events in the past have suffered from severe after-effects such as depression and PTSD, which were identified as having a direct impact on parenting style. The results are consistent with studies by Chan [29] and Harrington & Dubowitz [30], who demonstrated a relationship between parental emotional problems and child abuse. Today, services for migrant and refugee families, including NK refugees, are mainly focused on economic support [31], but this study shows that emotional recovery services such as psychological treatment and counseling for past experiences and trauma are equally important.

This study determined that stress arising from the adaptation of NK refugee parents has a great effect on child abuse. This is similar to studies on immigration and refugees of Hispanic and Vietnamese communities [21,22] (p. 3). The results of this study show that the stress experienced by NK refugees during migration caused psychological problems like depression, helplessness, and aggression, which led to negative attitudes in parenting.

Moreover, NK refugee parents with patriarchal attitudes were concerned about losing their authority over their children who were quicker to adapt, which led to stricter discipline and corporal punishment. These results suggest that they need direct and active interventions such as anger control programs, stress relief programs, and cognitive-behavioral programs for NK refugee parents. In addition, providing self-help clubs and mentoring services with their South Korean neighbors could eliminate the sense of isolation and alienation, which, in turn, would then allow them to adapt better in South Korean society.

Finally, NK refugee parents showed low parenting efficacy due to confusion in their parenting style and lack of parenting information, and the low parenting efficacy was related to child abuse. This is consistent with studies by [32,33,34] Luster & Kain, Shin and Ahn, which identified that the lower the parenting efficacy, the more they were inclined to emphasize discipline and coercive parenting. The result of this study is similar to [35] Gross et al.’s study, in that the higher the efficacy of parenting, the more positive the parenting tended to be. Along with confusion about parenting, they tended to choose a parenting pattern that they learned in North Korea, a method that focused on physical punishment. This phenomenon is more often found in parents who have migrated from countries with a patriarchal culture and, as expected, it is also seen in parents of North Korean refugee families. So, in light of the results of this study, thinking seriously about their parenting role is a necessity. In other words, it seems that they need customized parenting education programs that can improve their efficacy.

The educational content should be focused on changing misunderstandings about the parenting role and disseminating useful parenting knowledge. Considering that they are NK refugees who lack the time to participate in educational programs due to low wages and job insecurity, other ways, such as offering incentives or providing online education, could also be helpful.

## 5. Limitations

This study lacked diversity among the subjects. The study was conducted with only those participants who expressed their willingness to participate, so parents who engage in unstable employment situations were excluded. Therefore, follow-up research should try to include various NK participants, including single parents and young parents who may have more difficulty raising their children. In addition, in-depth interviews were difficult in this study. In order to understand the details, additional questions such as the background of their defection, and information about family members remaining in the North are sometimes required, but there is a limitation as questions were difficult, considering the sensitivity of the questions and the participants’ emotions. In addition, if it was determined that there was an expected risk of information exposure, even if the information was voluntarily stated by participants, the issue was not actively addressed in this study.

## 6. Conclusions

This qualitative research found that the issue of child abuse by NK refugees’ parents arises in a specific context of migration. This is similar to studies of migrant and refugee families abroad. We suggest that service providers should consider the culture and experience of both the entry country and migrant country when intervening in their problem. Similarly, child abuse prevention services previously designed for non-refugee families present many limitations to NK refugee families. Therefore, the government, local governments, and related organizations should develop child abuse prevention programs that consider the specific needs of NK refugees.

Previous results show that child abuse is more likely to be caused by personal, emotional, cognitive, and environmental factors that are interrelated and, influencing each other, rather than being caused by a single factor. This conversely suggests that utilizing the ‘virtuous circle of resources’ for the prevention of abuse is possible. In other words, intervention in some factors may affect others since the factors are linked together. Therefore, it is necessary to consider the structural relationship and the size of influence of each risk factor when balancing realistic problems as they arise in practice, such as when prioritizing the service supply is necessary due to limited resources.

In addition, in this study, the mechanism of occurrence of abuse by case was found to be very specific, diverse, and contextual. Therefore, the intervention for preventing child abuse is expected to be more effective if service providers add to their efforts to closely assess the individual circumstances and context they are in.

## Figures and Tables

**Table 1:** Characteristics of study participants.

Participant	Gender	Age	Settlement Period	Number of Children	Characteristics
A	F	53	6	2	- Living with one son. Has a daughter left in North Korea. - Over-obsessed with child, Wants to control their child’s behaviors.
B	F	35	5	2	- Living with one son and one daughter. - The frequency and intensity of child abuse are serious. - Experienced extreme fear due to frequent life-threatening situations during the defection.
C	F	28	3	2	- Living with one daughter and one son. - Severe corporal punishment of children due to the influence of growing up in a strict family environment. - Has experienced a lot of discrimination in South Korean society and high adaptation stress.
D	F	46	4	1	- Living with one son. - Having difficulty communicating with children and threatens them whenever they are angry. - Was caught by NK secret police officers and severely tortured and interrogated in NK detention.
E	F	40	2	1	- Living with two daughters. - Conflicts with children are serious and often threatens children. - Being forced to marry a Chinese man for human trafficking and experienced severe domestic violence by her husband.

**Table 2:** Research results.

Meta Themes	Sub-Themes	Parenting Patterns and Types
The Weakness of Family Functionality	(1) Unstable parent-child attachment due to long separation	Decreasing family attachment and closeness
	(2) Emotional problems of parents caused by traumatic event	Physical violence and neglect due to aggression, lethargy, etc.
Cultural Adaptation Stress in a Strange Society	(1) Stress transfer and display to children during child-rearing process	Venting anger, excessive interference, and control over children
	(2) Family conflicts due to differences in adaptation levels between parents and children	Strict discipline and corporal punishment to protect parental authority.
Degraded Parenting Effectiveness	(1) Confusion about the role of parents in the process of migration	Inconsistent parenting attitude
	(2) Lack of parenting information	Use of traditional parenting patterns (punishment-oriented parenting style)

## Data Availability

The data presented in this study are available on request from the corresponding author. The data are not publicly available due to Participant’s personal information.
